# Efficacy and safety of early ultrafiltration in patients with acute decompensated heart failure: a meta-analysis

**DOI:** 10.3389/fcvm.2023.1234092

**Published:** 2023-10-18

**Authors:** Shuai Chen, Hongqi Wang, Bin Ning

**Affiliations:** Department of Cardiovascular Medicine, Fuyang People’s Hospital Affiliated to Anhui Medical University, Fuyang, China

**Keywords:** efficacy, safety, early ultrafiltration, acute decompensated heart failure (ADHF), meta-analysis

## Abstract

**Objective:**

The objective of this study is to investigate the efficacy and safety of early ultrafiltration in patients with acute decompensated heart failure.

**Methods:**

A systematic search was conducted on PubMed, Cochrane Library, and EMbase databases from inception to April 2023 to identify randomized controlled trials that compared the efficacy and safety of early ultrafiltration and conventional diuretics in patients with acute decompensated heart failure. Two investigators independently screened all eligible studies and extracted relevant data. The primary outcomes of interest were changes in body weight and creatinine levels, as well as the rate of readmission and mortality within 30 days. Meta-analysis was conducted using RevMan 5.4 software.

**Results:**

This meta-analysis included eight studies and found that early ultrafiltration was effective in reducing body weight in patients with acute decompensated heart failure (*RR* = 1.45, 95% CI: 0.54–2.35, *P* = 0.002), but it also increased serum creatinine (*RR* = 0.1, 95% CI: 0.03–0.17, *P* = 0.003). However, it did not reduce the 30-day rehospitalization rate or mortality rate (30-day rehospitalization rate: *RR* = 0.84, 95% CI: 0.62–1.14, *P* = 0.28; Mortality: *RR *= 0.90, 95% CI: 0.57–1.44, *P* = 0.67).

**Conclusion:**

Although early ultrafiltration is more effective in reducing body weight in patients with acute decompensated heart failure, it is associated with an increase in serum creatinine levels and does not reduce the rate of readmission or mortality within 30 days.

**Systematic Review Registration:**

identifier: CRD42023416152.

## 1. Introduction

Heart failure (HF) is a critical aspect of cardiovascular disease, with device therapy, including ultrafiltration therapy, emerging as a viable option in recent years. Ultrafiltration is a treatment method that involves the passage of water and small to medium weight solutes through a semi-permeable membrane, thereby reducing the volume load (1). Both Chinese and European guidelines recommend ultrafiltration for heart failure patients with significant volume overload and poor response to conventional diuretics to quickly relieve symptoms of heart failure and fluid retention (2). The ACC/AHA guidelines are more active in recommending ultrafiltration therapy and do not emphasize diuretic resistance (3). They also consider significant fluid retention as an indication of ultrafiltration. The selection of the optimal timing for ultrafiltration is still a topic of discussion. This study aims to examine the effectiveness and safety of early ultrafiltration for patients with acute decompensated heart failure through a meta-analysis of randomized controlled trials.

## 2. Materials and methods

### 2.1. Inclusion and exclusion criteria

#### 2.1.1. Study types

Randomized controlled trials.

#### 2.1.2. Subjects

This study included patients of any race, nationality, gender, and stage of disease who had been clinically diagnosed with acute decompensated heart failure.

#### 2.1.3. Interventions

The study compared the effectiveness of two treatment methods: early ultrafiltration and traditional diuretics.

#### 2.1.4. Outcome measures

The primary outcome measures were changes in body weight and creatinine, rates of rehospitalization within 30 days, and mortality.

#### 2.1.5. Exclusion criteria

(1) ultrafiltration therapy was not administered within 24 h after admission; (2) animal experiments, case reports and repeated published studies; (3) studies with incomplete or unextractable data; (4) non-English literature.

### 2.2. Literature search

A systematic search was conducted on PubMed, Cochrane Library, and EMbase databases using the following search terms: ((cardi* or heart* or myocard*) and (fail* or incompet* or insufficien* or decomp*)) and (Ultrafiltration or ultrafiltration or UF). The search was limited to the period from the inception of the databases to April 2023.

### 2.3. Literature screening and data extraction

The literature was screened and data was extracted and cross-checked by two independent researchers. Any disagreements were resolved through discussion. The screening process involved reading the title first and then excluding any obviously irrelevant literature. After this, the full text of remaining articles was read to determine if they were relevant. In cases where additional information was needed, the original authors were contacted by mail or telephone. The extracted data included the first author, year of publication, country, number of cases, sex, age, comorbidities, changes in body weight and creatinine, rate of readmission within 30 days, and mortality.

### 2.4. Assessment of the risk of bias of the included studies

The included studies were evaluated for risk of bias by two independent researchers and their results were cross-checked. The Cochrane Risk of Bias tool was used for this assessment.

### 2.5. Statistical analysis

Meta-analysis was performed using Rev Man 5.4 software. RR was used as the effect index for count data, while MD was used as the effect index for measurement data. The point estimate and 95% CI were reported for each effect size. Heterogeneity among the included studies was assessed using a *χ*^2^ test (test level *α* = 0.1) and quantitatively determined by combining with *I*^2^. After excluding the influence of obvious clinical heterogeneity, a random-effects model was used for the meta-analysis.

## 3. Results

### 3.1. Literature screening process and results

**Figure 1:**
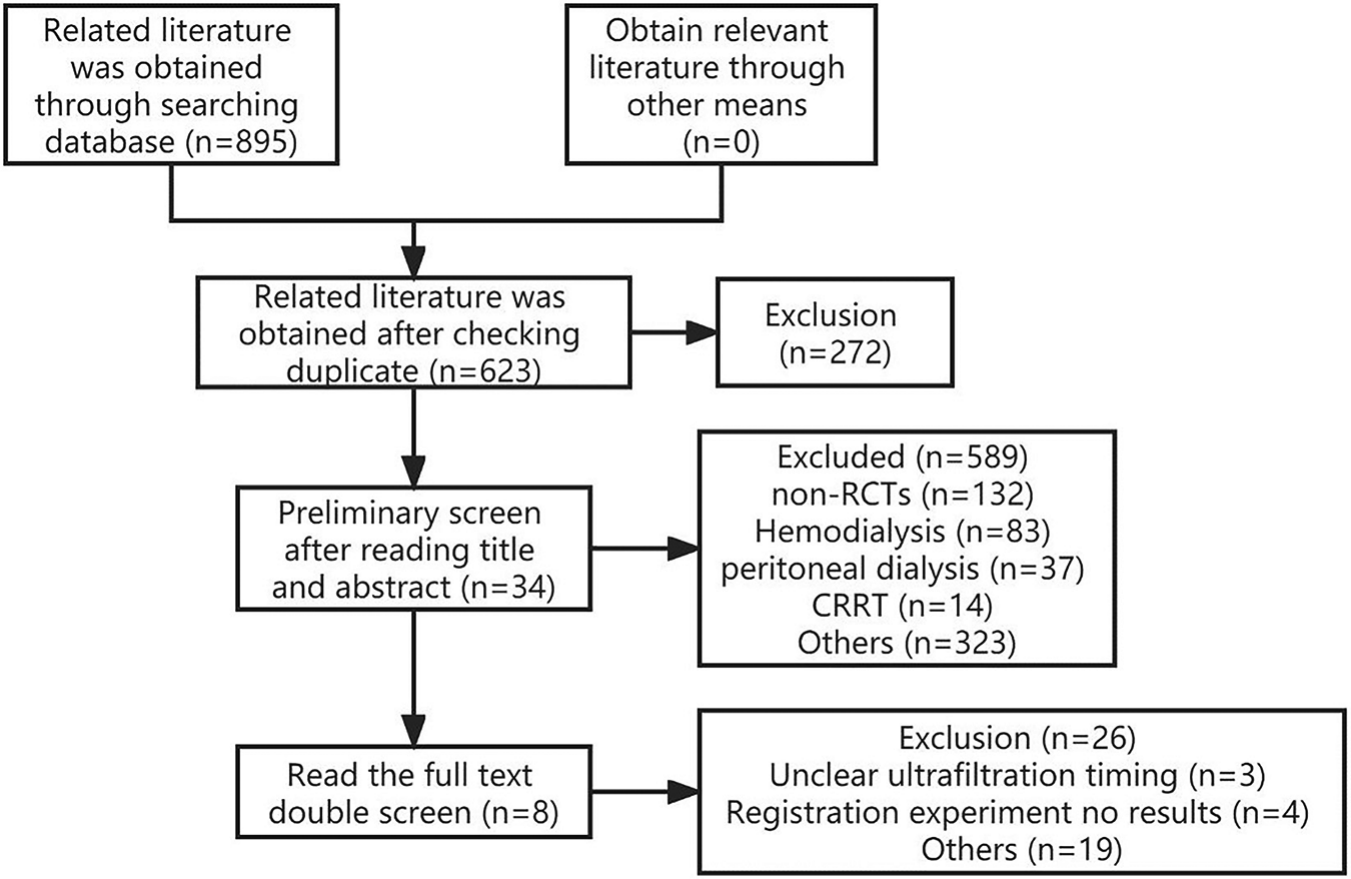
Literature screening process and results.

A total of 894 relevant literatures were retrieved and underwent a layer-by-layer screening process. Ultimately, 8 studies (4–11) were included. The literature screening process and results are illustrated in Figure 1.

### 3.2. Basic characteristics of included studies and results of bias risk assessment

**Figure 2:**
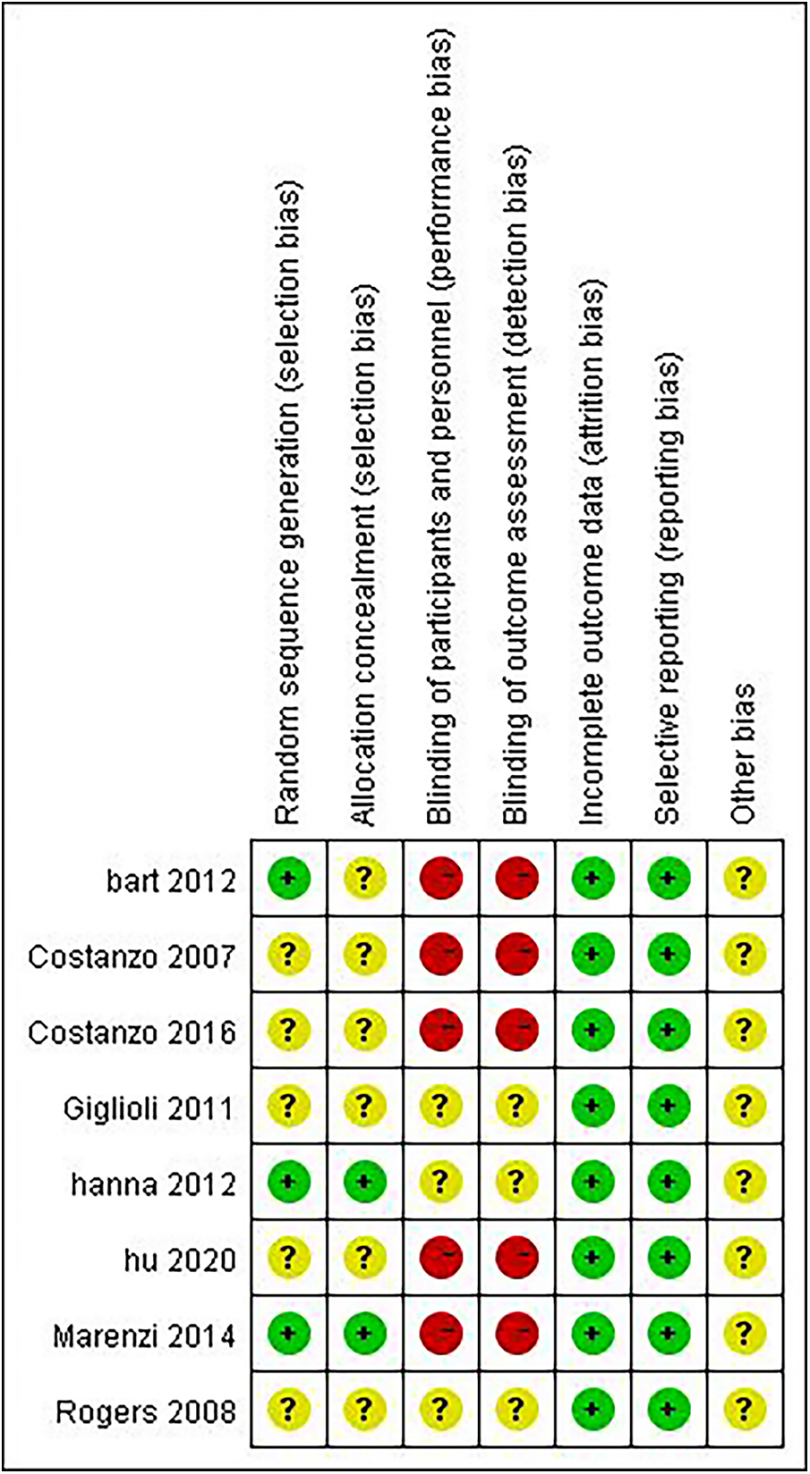
Results of bias risk assessment of the included studies.

The basic characteristics of the included studies are shown in Table 1, and the risk of bias assessment results of the included studies are shown in Figure 2.

**Table 1:** Basic characteristics of the included studies.

Study	Country	Cases	Male %	Age (year)	Hypertension %	DM %
Rogers et al. (4)	USA	19	78/60	64 ± 15/54 ± 16	78/40	78/50
Costanzo et al. (5)	USA	221	69.1/73	67 ± 13/67 ± 13	88.2/83	61.8/64
Marenzi et al. (6)	Italy	56	83/81	73 ± 9/75 ± 8	66/48	45/59
Giglioli et al. (7)	Italy	30	87/87	72.4 ± 14.1/65.8 ± 18.4	20/60	40/60
Hu et al. (8)	China	100	55/55	70.6 ± 10.44/73.52 ± 9.83	80/80	65/63.3
Hanna et al. (9)	USA	36	84.2/76	60 ± 9.1/59 ± 15.5	42.1/52.9	36.8/29.4
Bart et al. (10)	USA	188	72/78	66 (57–78)/69 (61–78)	–	67/65
Costanzo et al. (11)	USA	200	70/68	62 ± 15/63 ± 14	74/74	50/49

### 3.3. Results of meta-analysis

**Figure 3:**
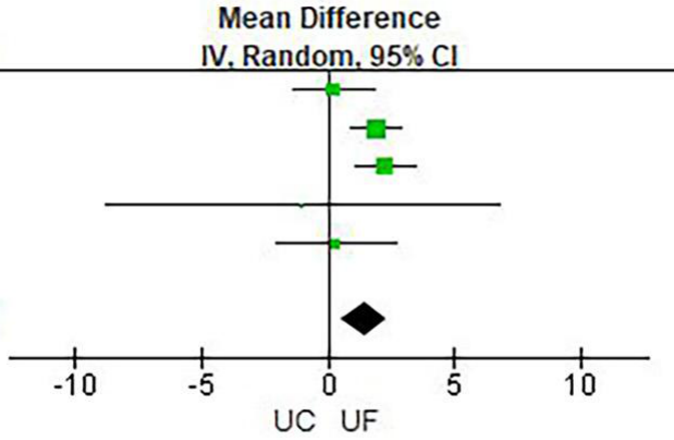
Weight change.

**Figure 4:**
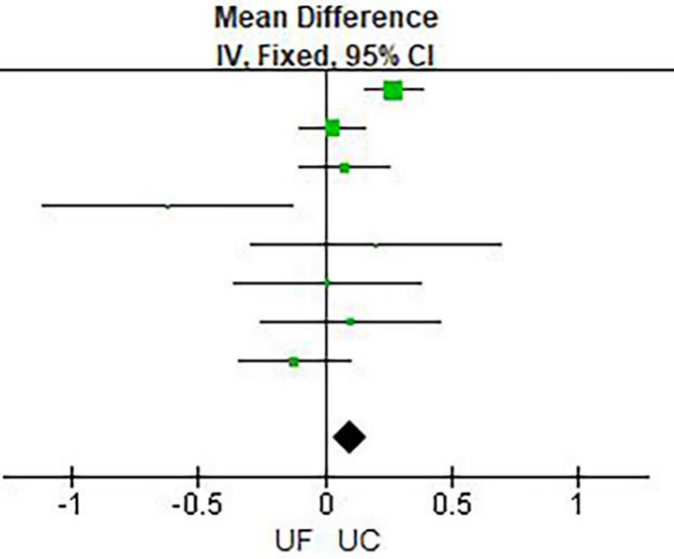
Creatinine change.

**Figure 5:**
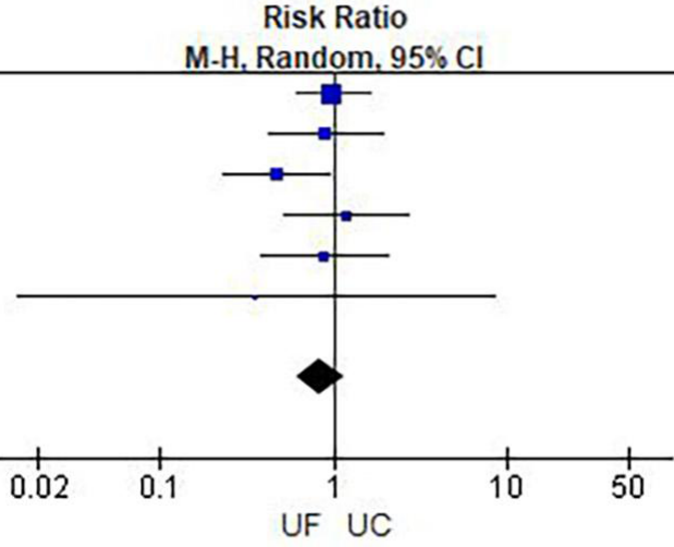
Rehospitalization rate within 30 days.

**Figure 6:**
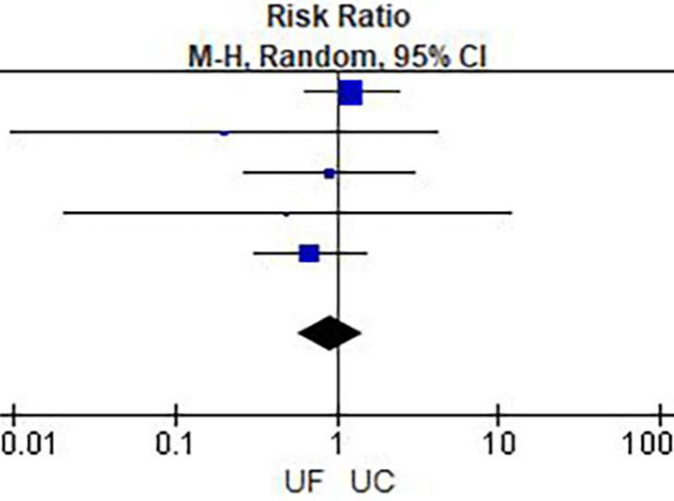
Mortality.

Results of meta-analysis are presented in Table 2 and Figures 3–6. This meta-analysis found that early ultrafiltration was effective in reducing body weight in patients with acute decompensated heart failure (*RR* = 1.45, 95% CI: 0.54–2.35, *P* = 0.002), but it also increased serum creatinine (*RR* = 0.1, 95% CI: 0.03–0.17, *P* = 0.003). However, it did not reduce the 30-day rehospitalization rate or mortality rate (30-day rehospitalization rate: *RR* = 0.84, 95% CI: 0.62–1.14, *P* = 0.28; Mortality: *RR* = 0.90, 95% CI: 0.57–1.44, *P* = 0.67).

**Table 2:** Changes in body weight, changes in creatinine, readmission rates within 30 days and mortality.

Name	Statistical method	Effect measure	Analysis model	Mean 1	SD 1	Total 1	Mean 2	SD 2	Total 2	Effect Estimate	CI Start	CI End	Weight
UF VS UC													
Body weight	IV	Mean difference	Random			251			277	1.445983	0.538299	2.353666	100
Bartet al. (10)				5.67	4.84	92	5.45	6.46	94	0.22	−1.41816	1.858159	20.7398
Costanzo et al. (11)				5	3.16	83	3.1	3.51	84	1.9	0.887292	2.912708	35.59658
Hu et al. (8)				2.94	3.76	40	0.64	0.91	60	2.3	1.112253	3.487747	30.518
Marenzi et al. (6)				7	12.5	27	8	17	29	−1	−8.779	6.778997	1.333117
Rogers et al. (4)				2.2	2.6	9	1.9	2.7	10	0.3	−2.08449	2.684489	11.8125
Creatinine	IV	Mean difference	Fixed			378			407	0.101736	0.033457	0.170015	100
Bart et al. (10)				0.23	0.44	92	−0.04	0.35	94	0.27	0.155589	0.384411	35.61537
Costanzo et al. (11)				0.1	0.42	69	0.07	0.35	75	0.03	−0.09687	0.156867	28.96539
Costanzo et al. (5)				0.13	0.88	107	0.05	0.3	107	0.08	−0.09616	0.256163	15.02268
Giglioli et al. (7)				−0.55	0.75	15	0.07	0.63	15	−0.62	−1.11568	−0.12432	1.897448
Hanna et al. (9)				0.2	0.75	19	0	0.75	17	0.2	−0.29075	0.690749	1.935779
Hu et al. (8)				−0.01	0.92	40	−0.02	0.9	60	0.01	−0.35489	0.37489	3.501465
Marenzi et al. (6)				0.1	0.66	27	0	0.7	29	0.1	−0.25621	0.456206	3.674272
Rogers et al. (4)				−0.01	0.31	9	0.11	0.15	10	−0.12	−0.34285	0.102849	9.387598
Readmission	MH	Risk ratio	Random			369			393	0.844839	0.62359	1.144588	100
Bart et al. (10)				0	0	90	0	0	93	0.990278	0.604827	1.621374	37.93041
Costanzo et al. (11)				0	0	88	0	0	86	0.895833	0.41801	1.919853	15.86952
Costanzo et al. (5)				0	0	105	0	0	108	0.467532	0.232735	0.939209	18.94885
Hanna et al. (9)				0	0	19	0	0	17	1.192982	0.519144	2.741451	13.31923
Hu et al. (8)				0	0	40	0	0	60	0.875	0.37702	2.03073	13.00796
Marenzi et al. (6)				0	0	27	0	0	29	0.357143	0.015169	8.408812	0.92403
Mortality	MH	Risk ratio	Random			290			311	0.904188	0.569691	1.435088	100
Bart et al. (10)				0	0	94	0	0	94	1.230769	0.627477	2.414101	47.01773
Costanzo et al. (5)				0	0	110	0	0	111	0.201802	0.009799	4.155886	2.332004
Hanna et al. (9)				0	0	19	0	0	17	0.894737	0.263702	3.035833	14.29688
Hu et al. (8)				0	0	40	0	0	60	0.495935	0.020706	11.87848	2.115471
Marenzi et al. (6)				0	0	27	0	0	29	0.683502	0.310368	1.505227	34.23791

## 4. Discussion

The study revealed that early ultrafiltration was more effective in reducing body weight in patients with acute decompensated heart failure. However, it caused an increase in creatinine and did not reduce the rate of readmission or mortality within 30 days.

It should be noted that, diuretics have a poor dose-effect relationship and cannot accurately predict urine volume. Many clinical trials have shown that ultrafiltration therapy is safe and effective (12). Ultrafiltration therapy has distinct advantages in managing fluid overload. It facilitates the removal of body fluids, excretes a higher amount of total sodium, and has favorable hemodynamic effects. Additionally, this therapy does not cause electrolyte disturbances or activate the neuroendocrine system. Moreover, it can restore the diuretic effect in some patients who have not responded to other diuretic treatments (13). Extracorporeal ultrafiltration can be utilized for mechanical dehydration, with the amount and speed of dehydration being determined based on the patient’s fluid load. Typically, the initial blood pump flow during treatment is set between 20 and 30 ml/min. It is important to note that a higher flow rate can increase the cardiac load and should not exceed 50 ml/min. The ultrafiltration speed is usually set at 200 to 300 ml/h, with a maximum limit of 500 ml/h. This speed can be adjusted according to the patient’s treatment response and vital signs. It is generally recommended that the total amount of ultrafiltration should not exceed 5,000 ml within a 24-h period. However, if the patient’s blood excitation dynamics are stable, the amount of ultrafiltration fluid can be increased based on the patient’s actual condition. However, ultrafiltration may lead to renal hypoperfusion, which can negatively impact renal function. Patarroyo et al. (14) demonstrated that while early ultrafiltration improved hemodynamics, it was linked to an increased risk of subsequent CRRT and higher in-hospital mortality rates. Other studies (15–17) have also indicated that worsening renal function in heart failure patients is associated with a poor prognosis.

According to the Heart Failure Registry study, it was found that out of 2,067 patients with heart failure, 21% of them developed diuretic resistance while undergoing treatment (18). Recent studies (19, 20) suggest starting ultrafiltration therapy early in patients with CHF, even before diuretic therapy becomes ineffective. This is particularly beneficial for patients with severe dyspnea symptoms of left heart failure, as ultrafiltration can effectively and regularly remove excess body fluid and improve symptoms rapidly (21), providing more reliable results than diuretics. Additionally, early use of ultrafiltration can buy time for further treatment. However, in cases of refractory heart failure or severe cardiorenal syndrome, ultrafiltration may not be effective as a “rescue” treatment. The RAPID-CHF test initiated ultrafiltration treatment within 24 h of admission for patients with acute decompensated heart failure (ADHF). The average volume of fluid removed through ultrafiltration in a 24-h period was 4,650 ml, compared to 2,838 ml in the diuretic group. This indicates that ultrafiltration was more effective than diuretic treatment in terms of fluid clearance (19). The UNLOAD study, which is the largest randomized controlled trial on ultrafiltration therapy for ADHF, divided 200 patients into an early ultrafiltration group and a routine diuresis group. The results showed that patients in the early ultrafiltration group experienced greater weight reduction, a lower rate of re-hospitalization within 90 days, similar relief of dyspnea compared to the routine diuresis group, and a lower incidence of hypokalemia (11). Previous meta-analyses (22) have compared ultrafiltration with diuresis in patients with heart failure and have shown that ultrafiltration is more effective in reducing volume load and rehospitalization rate, but it does not reduce the incidence of adverse events and mortality. This study’s innovation lies in the timing of early ultrafiltration, which aims to determine the efficacy and safety of this approach in patients with acute decompensated heart failure. This study has some limitations that should be considered. Firstly, the sample size of 8 studies included in this research was relatively small, which may have limited the power of the evidence provided. Secondly, there was heterogeneity among the results of the different studies included, which could be due to various confounding factors, such as differences in sample sizes.

Based on current evidence, early ultrafiltration appears to be more effective in reducing body weight in patients with acute decompensated heart failure. However, it is important to note that this treatment may increase serum creatinine levels and does not necessarily reduce the rate of readmission or mortality within 30 days. It is important to acknowledge that the conclusions drawn from the available studies are limited by both the quantity and quality of the research. As such, further high-quality studies are needed to verify these findings.

## Data Availability

The original contributions presented in the study are included in the article/Supplementary Material, further inquiries can be directed to the corresponding author.
